# Use of Intravenous Tranexamic Acid in Patients Undergoing Plastic Surgery: Implications and Recommendations per a Systematic Review and Meta-Analysis

**DOI:** 10.7759/cureus.62482

**Published:** 2024-06-16

**Authors:** Christopher R Meretsky, Andreas Polychronis, Anthony T Schiuma

**Affiliations:** 1 Surgery, St. George's University School of Medicine, New York, USA; 2 General Surgery, St. George's University School of Medicine, New York, USA; 3 Orthopedic Surgery, Holy Cross Hospital, Fort Lauderdale, USA

**Keywords:** tbl, ecchymosis, edema, blood loss, blepharoplasty, rhinoplasty surgery, plastic surgery, intravenous tranexamic acid

## Abstract

With increasing interest in aesthetic plastic procedures, the event of blood loss has compromised patients' safety and satisfaction. Tranexamic acid (TXA) is a drug used for the reduction of blood loss during surgical procedures. This systematic review aims to evaluate the clinical efficacy and safety of TXA in aesthetic plastic surgery for the reduction of bleeding and related complications. The Preferred Reporting Items for Systematic Reviews and Meta-Analyses (PRISMA) guidelines were followed. Electronic databases PubMed, EMBASE, Cochrane Library, and Google Scholar were searched. The medical subject headings (MeSH) keywords used for data extraction were (“TXA,” OR “tranexamic acid,”) AND (“plastic surgery,” OR “aesthetic surgery,” OR “rhinoplasty,” OR “blepharoplasty,”) AND ("blood loss" OR "bleeding" OR "TBL") AND ("Edema" OR "ecchymosis”). A combination of these MeSH terms was used in the literature search. The timeline of research was set from 2015 to January 2024. A total of 7380 research articles were identified from the above-mentioned databases, and only 13 research articles met the inclusion criteria. There was a significant difference in total blood loss (TBL) among patients who had undergone plastic surgery procedures while on TXA as compared to a placebo (mean difference = -6.02; Cl: -1.07 to -0.16; p > 0.00001), and heterogeneity was found (degrees of freedom (df) = 9; I2 = 97%). Only two studies reported the average ecchymosis scores after TXA among interventions in comparison to the placebo group. This review provides evidence that TXA lowers TBL, ecchymosis, edema, and anemia during cosmetic surgery without significantly increasing thromboembolic consequences.

## Introduction and background

In recent decades, the popularity of aesthetic plastic surgery has grown rapidly, as 2.3 million cosmetic surgical procedures have been conducted during 2020 only [1]. The popularity of facial rejuvenation has increased drastically due to the ease in accessibility of cosmetic treatments, increased awareness, and reduced downtime as consumer demands. Self-improvement has been emphasized among present generations, and plastic surgical procedures have proved to be effective in this context [2]. According to statistics, about 20 million cosmetic procedures were performed globally by 2015 [3]. Another report shows that 14.9 million surgical procedures were performed by plastic surgeons in 2022, and overall, an 11.2% increase in the number of procedures was reported, showing a steady increase in some cosmetic surgeries over the last four years.

Typically, these surgeries are performed with careful consideration of safety profiles, and acceptable risks and relatively healthy patients are chosen in controlled settings [4,5]. However, several known complications are associated with aesthetic plastic surgical procedures, including blood loss and bleeding that lead to anemia and hematoma formation. Additionally, these safety concerns related to bruising and excessive blood loss during aesthetic surgeries can lead to decreased patient satisfaction [6]. Kaoutzanis et al. [7] conducted a prospective study on 129,007 patients of cosmetic surgery, and a major event among those was a hematoma, which affected 1% of the total study population. Every year, millions of plastic surgery patients experience bleeding issues, even at modest rates. For this reason, it is critical to search for supplements that may further minimize problems to enhance patient safety and cosmetic results. 

In aesthetic facial plastic surgery, hemostasis is a critical event. Another major event after intraoperative blood loss is an increased need for transfusion and operation time, which can lead to morbidity and chances of complications [8]. Frequent consequences of aesthetic facial plastic surgery include postoperative edema and inflammation, which cause patients additional psychological stress and may limit their ability to socialize while they heal. Additionally, edema and ecchymosis impair one’s capacity to evaluate surgical outcomes both during and after surgery [9]. Excessive bleeding emphasizes the need for allogenic transfusion but can be accommodated by blood product transfusion, which can be a life-saving measure and restore patient hemodynamic parameters [10]. However, blood product transfusion is associated with several non-infective and infectious complications, leading to an increase in mortality and morbidity. Therefore, reducing perioperative bleeding during aesthetic surgeries is crucial to improving overall survival and reducing several unwanted complications [11].

In plastic surgical procedures, there is a need to maintain a balance between fibrinolysis and coagulation that would prevent bleeding and maintain circulation. Despite appropriately maintained hemostasis, excessive bleeding may occur due to coagulation resulting from fibrinolysis [12]. In soft-tissue operations such as aesthetic plastic surgical procedures, bleeding can be mostly controlled by using cautery, careful surgical technique, and local adrenaline filling of the wound edges. However, bone tissue cannot be penetrated or managed to the same degree. Consequently, techniques including hypotensive anesthesia and pharmaceutical assistance are employed to manage bleeding in bone tissue [13]. 

Numerous treatments have been reported to decrease surgical bleeding during cosmetic procedures. The most effective interventions used for preventing surgical bleeding include E-aminocaproic acid [14], antifibrinolytic agent tranexamic acid (TXA) [15], and aprotinin. These surgical specialties mostly need antifibrinolytics to lessen the requirement for transfusions. These anti-fibrinolytic interventions increase clot stability and prevent fibrinolysis [16]. 

One possible medication to reduce bleeding and hematoma formation in individuals undergoing cosmetic plastic surgery is TXA. Tranexamic acid is a synthetic lysine analog and low-cost antifibrinolytic drug that inhibits the regulation of plasminogen to plasmin by binding it to lysine residues. It has anti-inflammatory effects due to inhibition of plasmin formation, as plasmin causes several inflammatory activities [17,18].

Moreover, the drug prevents the dissolution of fibrin clots by plasmin and blockage of plasmin-induced platelets that preserve platelets for subsequent clot formation. This drug was synthesized and approved for oral as well as intravenous use, but the use of systemic prophylactic decreases the volume of blood loss [19]. About 30% to 40% of total blood product transfusion is reduced after using TXA. The effectiveness of TXA has been reported in cardiac surgery, obstetric surgery, orthopedic surgery, trauma surgery, and cosmetic plastic surgery [20]. 

In the case of cosmetic plastic surgical procedures, the use of TXA is somehow limited due to fewer events of blood loss. However, the importance and role of TXA in maintaining patient satisfaction and safety cannot be ignored [21]. Multiple systematic reviews and meta-analyses have proved that TXA has a major role in the reduction of intraoperative bleeding, risks of thromboembolism or renal failure, and ultimately resulting in fewer chances of transfusion during major aesthetic surgeries [22]. Although a larger number of studies have reported weak evidence related to prothrombotic effects, it resulted from TXA, as compared to the results of studies that opposed the use of TXA.

However, TXA proves to be a promising option for decreasing bleeding and bleeding-linked complications in aesthetic plastic surgeries [23]. Moreover, several studies have been published on the effectiveness of TXA in spine surgery and cardiac and orthopedic surgeries, but few investigations exist in the field of cosmetic plastic surgery as limited blood loss events occur during these procedures [24-26]. Even less information or trials have been reported related to the efficacy of TXA in facial rejuvenation plastic surgical procedures. There is a lack of literature related to the clinical implications of TXA as standard practice [27]. Therefore, a systematic review was undertaken to evaluate the clinical efficacy and safety of TXA in aesthetic plastic surgery for the reduction of bleeding and related complications. The findings of this recent systematic review and meta-analysis on the clinical implications of TXA will help patient safety and satisfaction in aesthetic plastic surgery.

## Review

Methods

Study Design

The Preferred Reporting Items for Systematic Review and Meta-Analyses (PRISMA) guidelines were followed [1]. No additional ethical review was required, as this study was based on a meta-analysis of already published randomized controlled trials (RCTs) and retrospective and prospective studies.

Selection Criteria

The selection and screening of research articles were conducted per PRISMA guidelines [28]. The predefined selection criteria helped in the screening of research articles. Inclusion criteria: Retrospective and prospective studies or RCTs involving patients undergoing different types of plastic surgery; studies involving the implication of the drug TXA 3); studies that discuss outcomes of TBL, response rates, adverse events, edema, hematoma rates, adverse events, and average ecchymosis score; studies based on RCTs and prospective or retrospective cohort studies; and studies with full text and published in English.

Exclusion criteria: Studies that discuss the study population undergoing surgical treatments rather than plastic surgery, involve treatment strategies other than TXA, and discuss other outcomes than TBL, adverse events, and edema; already published systematic reviews, meta-analyses, scoping reviews, literature reviews, conferences, and case studies; and studies with non-full-text papers, duplicated publications, and published in languages other than English.

Search Strategy

**Figure 1:**
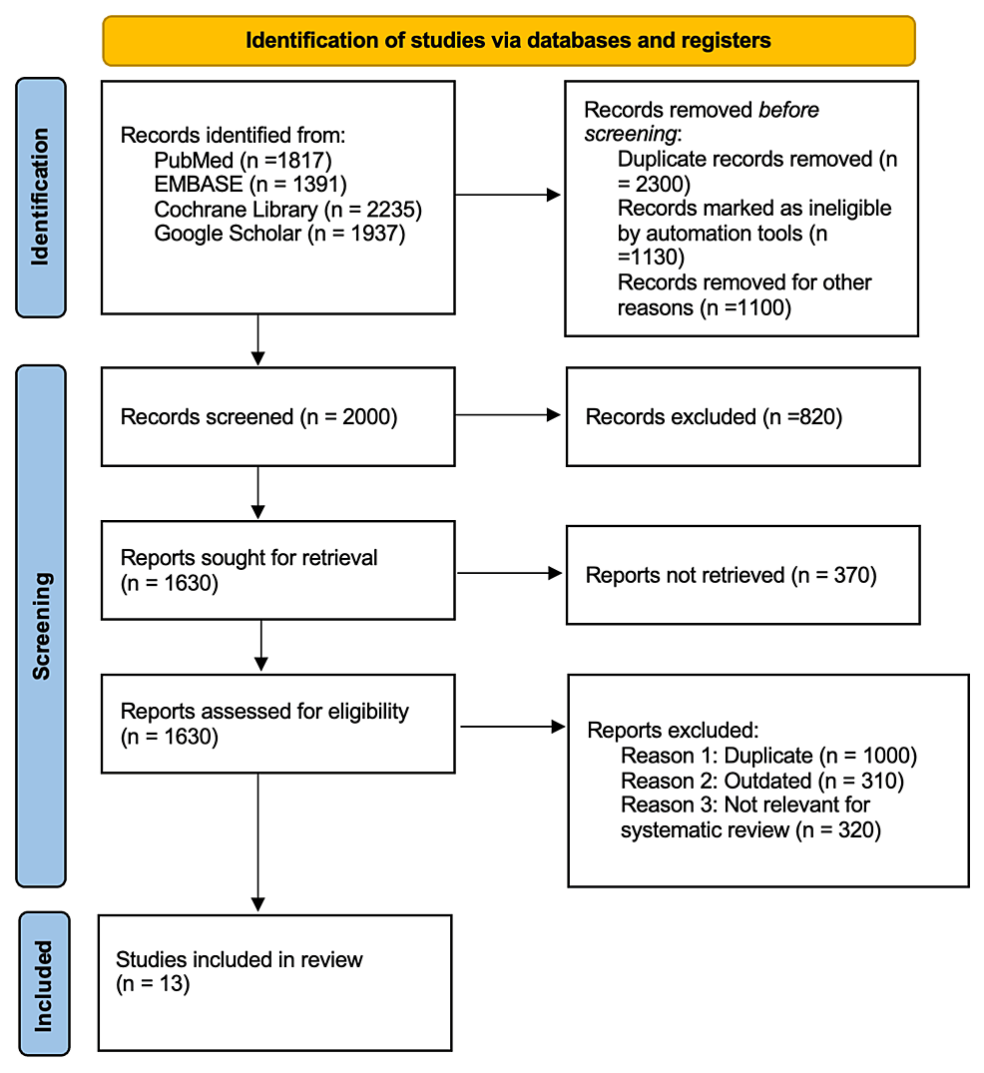
PRISMA flowchart of literature search and study selection PRISMA: Preferred Reporting Items for Systematic Reviews and Meta-Analyses

The research articles related to the study aims, i.e., the 'Use of Intravenous tranexamic acid in patients undergoing plastic surgery: safety recommendation through systematic review and meta-analysis' were extracted from different databases by following PRISMA guidelines [29]. Electronic databases such as PubMed, EMBASE, Cochrane Library, and Google Scholar were used for the search. The medical subject headings (MeSH) keywords used for data extraction were (“TXA,” OR “tranexamic acid,”) AND (“plastic surgery,” OR “aesthetic surgery,” OR “rhinoplasty,” OR “blepharoplasty,”) AND ("blood loss" OR "bleeding" OR "TBL") AND ("Edema" OR "ecchymosis”). A combination of these MeSH terms was used in the literature search. The timeline of research was set from 2015 to January 2024. We carefully examined the reference lists of all the articles included and reviewed papers to seek further work. Articles published in peer-reviewed journals and relevant medical guidelines were included in the review. The study selection process is illustrated in Figure 1.

Data Extraction

Each recognized citation's titles and abstracts were evaluated separately by two reviewers. Full-text papers were arranged and assessed with the eligibility requirements. Discussions were used to settle any disputes. Each reviewer extracted data independently from each paper included and tabulated it into a spreadsheet. All data were tabulated onto a predefined word table. The information extracted for each article included the author's name, year of publication, study population, study design, gender, follow-up, dosage, and outcomes for appraisal and analysis.

Risk of Bias Assessment

To evaluate the risk of bias of included RCTs, the Cochrane risk of bias assessment tool was used [30]. The bias was assessed based on five domains: (i) allocation concealment, (ii) selection bias or random sequence generation, (iii) performance bias or blinding of participants and personnel, (iv) detection bias or blinding of outcome assessment, (v) Selective bias or selective reporting, and other bias. Each domain's score was categorized into low risk, high risk, or unclear.

Study Outcomes

The primary outcomes studied in this systematic review and meta-analysis were TBL, length of hospital stay, average ecchymosis score, edema, hematoma rates, and adverse events.

Statistical Analysis

The pooled analysis was conducted by using the random effects of the Mantel-Haenszel methods [31]. The purpose of subgroup analysis was to evaluate the effects of intervention during follow-up. Moreover, the heterogeneity was measured by using the Q test and I2 statistics. If the I2 value was >50%, significant heterogeneity was considered. A significant difference was considered if the p-value > 0.05. The magnitude of heterogeneity was quantified by I2 statistics (mild, 0-30%; moderate, 31-50%; high, >50%). A p-value <0.05 was considered significant. Funnel and forest pooled estimates were reported to determine publication bias. The RevMan software version 5.4 (Cochrane Collaboration, London, UK) was used for pooled analysis. The analysis was done to evaluate the mean difference related to expected outcomes after antibiotics. 

Results

Search Strategy

About 7380 research articles were identified from the above-mentioned databases with 4560 duplicates related to the research title “Use of intravenous tranexamic acid in patients undergoing plastic surgery: implication recommendation through systematic review and meta-analysis” to fulfill research aims. About 2000 were retrieved after the removal of 820 articles. The primary screening of 1630 was conducted, and 370 research articles were excluded. The eligibility criteria were applied to 1630 research articles, and only 13 research articles met the inclusion criteria. All 1617 research articles were excluded due to screening and selection by PRISMA guidelines [32-46].

Risk Bias Assessment

**Figure 2:**
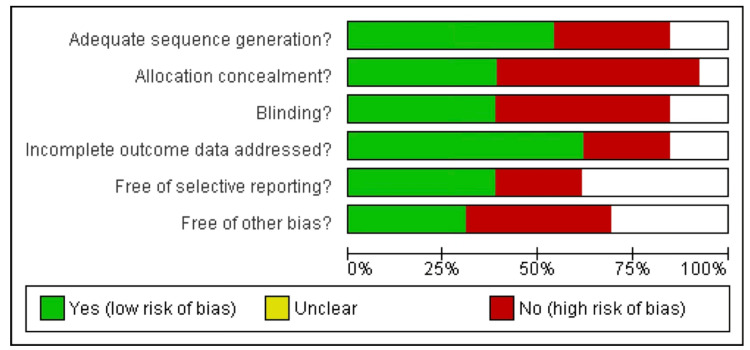
Risk bias graph of included studies

**Figure 3:**
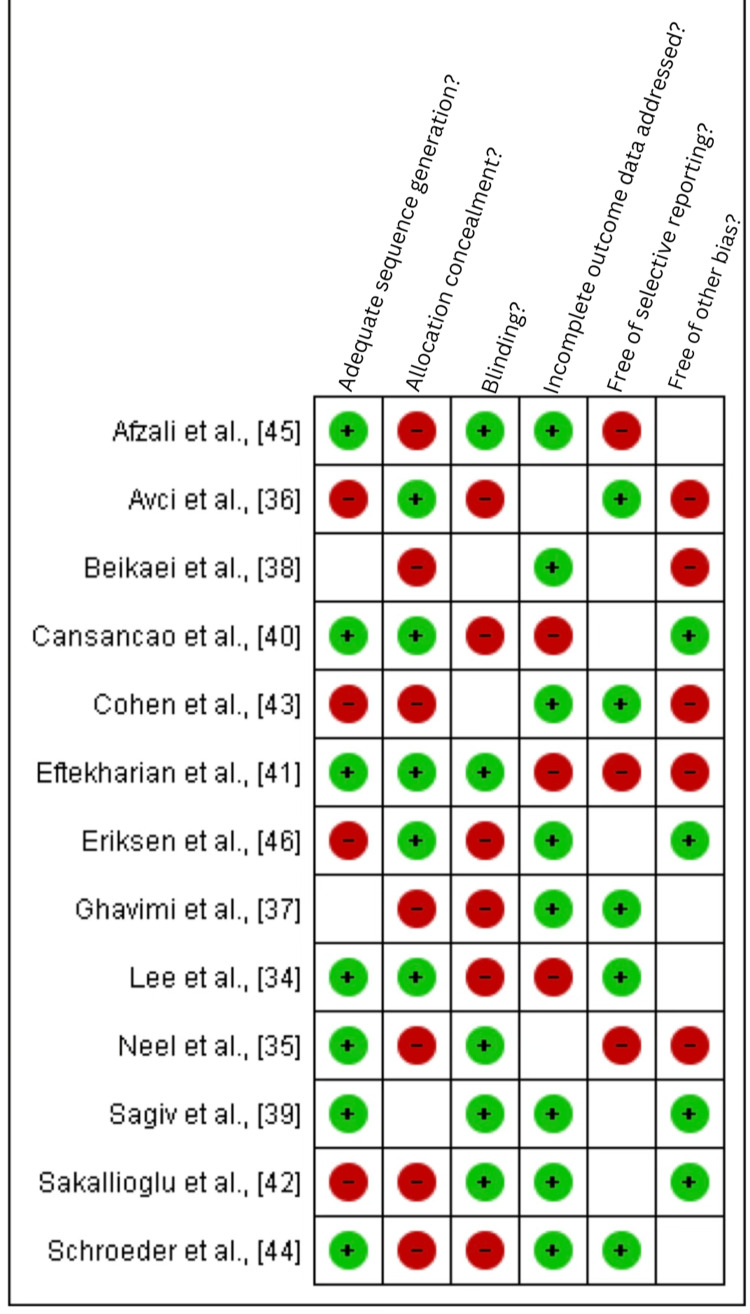
Summary of risk bias graph of included studies Red or minus: High-risk aspects of included studies, Green or plus: Low-risk aspects of included studies, Blank or white: Unclear [34-46]

The risk of bias [31] was summarized for each study (Figures 2-3). Only two studies were high risk, 10 studies were low risk, and two were moderate risk studies among the 13 included studies.

Characteristics of Included Studies

Through 13 included studies, about 1641 patients who underwent aesthetic plastic surgery have been discussed. To produce heterogeneity in results, these studies have been taken from different countries: four studies from Iran [37,38,41,45]; three studies from the USA [40,43,44]; two in Turkey [36,42]; and one in Singapore [34], one in Saudi Arabia [35], one in Israel [39], and one in Denmark [46], as mentioned in Table 1.

**Table 1:** Characteristics of included studies TBL: Total blood loss, TXA: Tranexamic acid, RCT: Randomized controlled trial

Author, Year	Study population	Gender	Country	Study design	Study follow up	Dose	TBL in mL	Average ecchymosis scores
Lee et al., 2016 [34]	561 patients	512 females and 49 males	Singapore	Retrospective cohort study	Two months			
Neel et al., 2023 [35]	425 patients: 181 patients received TXA, and 254 in control	374 females, 151 males	Saudia Arabia	Retrospective study	Six weeks	500 mg to 2 g		
Avci et al., 2020 [36]	90 patients: 60 in TXA, 30 in control group	76 females, 14 males	Turkey	RCT	One time	Intravenous 1 g to 2 g of TXA	TBL: 358.33 (128.07) in TXA and 372.22 (233.07) in control	
Ghavimi et al., 2017 [37]	60 patients: 30 in TXA and 30 in control		Iran	RCT	One time	10 mg/kg	TBL: 213 (65) mL in TXA and 254 (55) mL in control	Average ecchymosis score: 2.04 in TXA and 2.46 in control
Beikaei et al., 2015 [38]	96 patients: 48 in TXA and 48 in placebo	76 females, 20 males	Iran	Single-center, double-blind RCT	One time	10 mg/kg	TBL: 43.3±11.0 mL in the TXA and 60.3±9.5 mL in control	
Sagiv et al., 2018 [39]	34 patients: 17 in TXA and 17 in control	28 females, 6 males	Israel	Prospective pilot study	One time	10 mg/kg	TBL: 17.6 (5.7) in TXA and 19.1 (10.4) in control	Average ecchymosis score: 1.2 in TXA and 1.7 in control
Cansancao et al., 2018 [40]	20 patients: 10 in TXA and 10 in control	20 females	USA	Prospective, double-blind, non-randomized study	One time	10 mg/kg intravenous	TBL: 37.7 (15.5) in TXA and 59.5 (25.1) in control	
Eftekharian et al., 2016 [41]	50 patients: 25 in TXA and 25 in control	26 females, 24 males	Iran	Double-blind, randomized, clinical trial	One time	1 g (2 × 500 mg) oral use	TBL: 144.6 ± 60.28 mL in TXA and 199.6 ± 73.05 mL in control	
Sakallioglu et al., 2015 [42]	75 patients: 50 in experimental and 25 in control	62 females, 13 males	Turkey	Double-blind, randomized, clinical trial	One time	1g of TXA	TBL: 68 (21) in TXA and 133 (63) in control	
Cohen et al., 2021 [43]	44 patients: 27 in TXA and 17 in the control group	44 females	USA	RCT	One time	1 g dose of IV TXA	TBL: 1.74 (0.71) in TXA, 1.88 (0.78) in the control group	
Schroeder et al., 2020 [44]	76 patients: 44 in TXA, 32 in control		USA	Retrospective cohort study		1 g TXA	TBL: 14.8 cc with TXA, and 50.4 cc in the control group	
Afzali et al., 2022 [45]	80 patients: 50 in TXA, 30 in control		Iran	RCT	1 time	TXA 10 mg/kg	TBL: 11.99 in TXA, 19.19 in control	
Fenger-Eriksen et al., 2019 [46]	30 patients: 15 in TXA and 15 in control		Denmark	RCT	1 time	TXA 10 mg/kg	TBL: 18 ml in TXA, 52 ml in control	

Primary Outcomes

**Figure 4:**
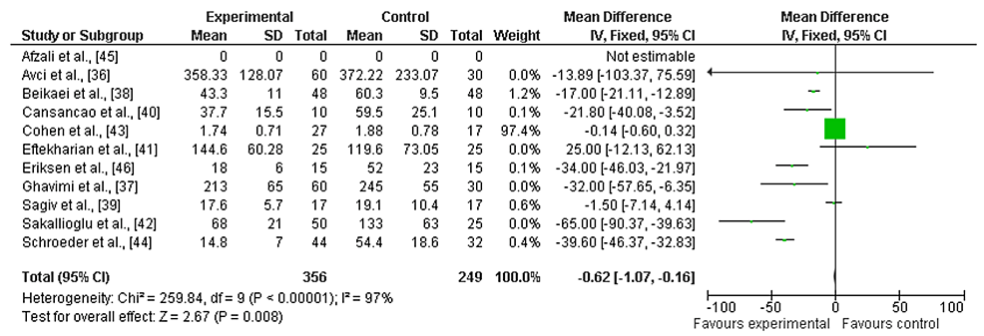
Forest plot of TBL among intervention vs. placebo group TBL: Total blood loss

**Figure 5:**
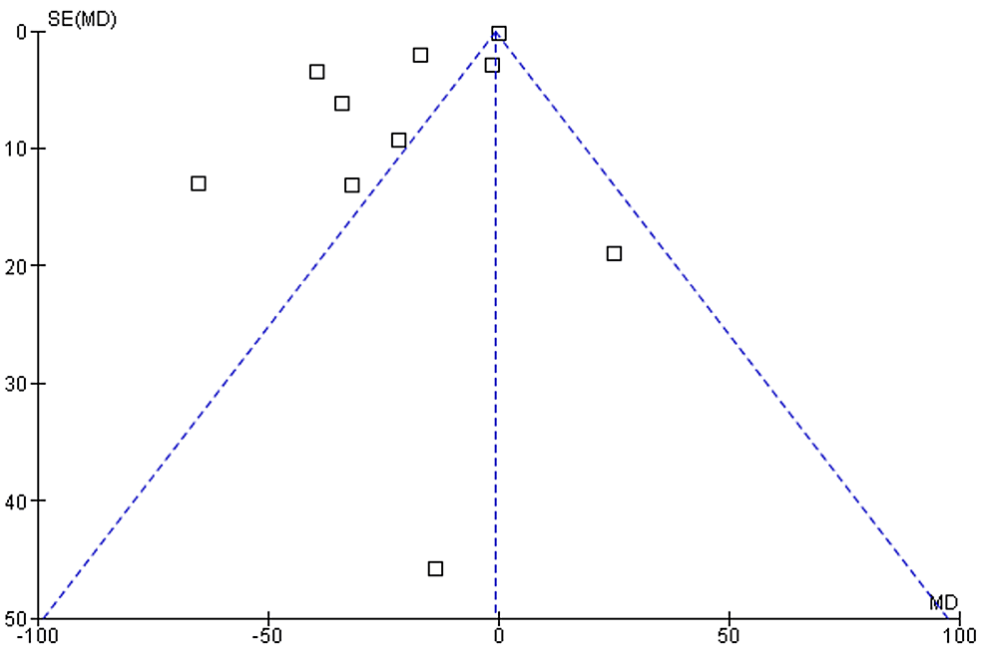
Funnel plot of TBL among intervention vs. placebo group TBL: Total blood loss, SE: Standard error

Total blood loss: Among 13 included studies, about 11 discussed the efficacy of TXA in reducing TBL after aesthetic plastic surgical procedures [36-46]. There was a significant difference in TBL among patients who had undergone the plastic surgical procedure as compared to a placebo (mean difference = -6.02; Cl: -1.07 to -0.16; p > 0.00001), and heterogeneity was found (degrees of freedom (df) = 9; I2 = 97%), as shown in Figure 4 and Figure 5.

Average ecchymosis score: The average ecchymosis scores measure the blood loss from broken capillaries into surrounding tissue. As in plastic surgical procedures, the average ecchymosis score measures the severity of bleeding from the surgical site. However, only two studies reported the average ecchymosis scores after TXA among interventions in comparison to the placebo group. 

Discussion

This systematic review and meta-analysis have been conducted to investigate the effectiveness of the TXA in reducing TBL, a main reason for anemia, and hematoma and ultimately leading to blood transfusion during aesthetic plastic surgical procedures. Eleven out of 13 studies included proved a significant mean difference in TBL among the group receiving TXA as compared to the placebo group [36-46]. However, other study outcomes were not reported among the included studies, so there is no outcome evaluated through software to evaluate the efficacy of TXA.

In plastic surgical procedures, excessive blood loss may occur, which leads to re-exploration, post-operative anemia, and hematoma, resulting in an emergent need for blood transfusion. Non-cardiac procedures such as cosmetic plastic surgery, blood transfusion, and anemia can lead to higher events of morbidity and mortality [47,48]. However, blood loss during different surgical procedures for aesthetic needs, such as rhinoplasty, blepharoplasty, and facial rejuvenation, is reported to be lower than other procedures. However, there is a need to involve interventions that can maintain patient safety and satisfaction during cosmetic surgical procedures [49].

Several interventions, such as TXA, have been extensively used for preventing perioperative blood loss. As described earlier, there are mainly three antifibrinolytic drugs: aprotinin, E-aminocaproic acid, and TXA. The last two are derivatives of the amino acid lysine that prevent blood clots from breaking down by binding to the plasminogen's lysine-binding site and blocking its transformation to plasmin, therefore inhibiting fibrinolysis. Additionally, they keep platelet receptors from being degraded by plasmin, maintaining platelet function [50]. 

In 1957, TXA was first manufactured for the management of hematoma and anemia, resulting from blood loss during surgical procedures. After that, the use of TXA has become common to prevent blood loss and result in blood product transfusion [51]. Tranexamic acid is about 10 times more effective than other amino acid lysine derivatives and binds tightly to plasminogen molecules. However, the effectiveness and safety of TXA are similar to other amino acid lysine derivatives. Therefore, TXA has been gaining much importance in several types of surgical procedures and is reported as the most used drug [52].

The implication of TXA has been well reported in several operations rather than plastic surgeries. The Clinical Randomization of an Antifibrinolytic in Significant Hemorrhage (CRASH)-2 trial reported the use of TXA in reducing blood loss, vascular occlusive events, linked morbidities, and rates of blood transfusion. This multi-centric randomized controlled trial involving 20,211 trauma patients reported TXA as an efficient drug for patients at risk of bleeding during or after surgical procedures [53]. They were randomized to either the TXA group or the placebo group within eight hours of the surgery. The findings demonstrated that early TXA administration decreased the incidence of bleeding-related death while showing no obvious rise in severe vascular occlusive incidents. Additionally, the CRASH-3 trial demonstrated that giving TXA within three hours of trauma decreased the number of fatal head injuries following brain damage with no signs of side effects or problems [54-57].

Several studies have been performed to investigate the efficacy of TXA in non-urgent surgeries, as Henry et al. [16] reported that TXA is effective in reducing bleeding during and after operations and chances of allogenic blood product transfusion by 1/3rd through 252 RCTs. Thus, TXA has reduced the morbidity and mortality rates resulting from blood loss during surgical procedures. Kagoma et al. [58] conducted another systematic review and meta-analysis to compare the effectiveness of TXA with placebo through 95 RCTs and found that TXA has reduced the probability of blood transfusion due to less blood loss.

However, the studies conducted to report the effectiveness of TXA use in aesthetic plastic surgeries are very limited. The trend of TXA implications in aesthetic plastic surgery has been now expanding. Tranexamic acid was first developed as a medication to stop severe bleeding and blood loss, but it is now used in cosmetic surgery to focus on a distinct set of results that are particularly significant for this patient population [59-61]. According to earlier systematic reviews assessing TXA in plastic surgery, it is effective in lowering blood loss and transfusion during orthognathic and cerebral vault rebuilding [62-65]. Rohrich et al. [66] reported the effectiveness of TXA and its potential to reduce bleeding, edema, and ecchymosis and increase patient safety and satisfaction through RCTs. However, the study used limited evidence to report the efficacy of TXA in aesthetic plastic surgeries. Brown et al. [64] reported recent evidence related to the use of TXA in aesthetic plastic surgeries and summarized that TXA is safe and effective in patients undergoing plastic surgery as it reduces blood loss and events of anemia and hematoma. 

According to our review, TXA can effectively reduce edema and postoperative ecchymosis after plastic surgery as well as ecchymosis following liposuction. Eleven trials showed a significant reduction in bleeding and blood loss, even though meta-analysis was not possible due to inconsistent measurements of ecchymosis and edema throughout the investigations. If bruising or edema are associated with TXA, more research on plastic surgery procedures and the use of a repeatable technique to measure ecchymosis and edema would be beneficial [65, 66].

This meta-analysis has used more recent research to evaluate the efficacy of TXA, as it is done only on plastic surgical procedures. We used the Cochrane Library tool to evaluate the methodological risk of bias to ensure the quality of included studies. The publication bias of included studies was robust, which ensured the quality of the meta-analyses. We conducted a pooled analysis of TBL only, ignoring hematoma rates and other primary outcomes. However, there are a few limitations that should be considered. First, a limited number of studies were available comparing TXA‘s efficacy with other treatment strategies. Second, all studies were retrospective studies or non-randomized controlled trials, as it should include randomized controlled trials to produce heterogeneity. Third, even though we used stringent inclusion and exclusion standards, we probably overlooked some additional biological elements that might have impacted the conception result. Fourth, we limited the scope of our analysis to publications that were composed in English.

## Conclusions

This meta-analysis and systematic review provide an overview of how TXA affects bleeding outcomes in cosmetic plastic surgery. This paper gives evidence that TXA lowers TBL, ecchymosis, edema, and anemia during cosmetic surgery without significantly increasing thromboembolic consequences. It is the most recent systematic review with meta-analysis assessing TBL. Considering the notable enhancement in bleeding outcomes for patients undergoing aesthetic surgery, it seems sensible to employ TXA more frequently in clinical settings. Nevertheless, it is not advised to use TXA in individuals with hematologic or coagulation abnormalities due to a lack of clinical evidence.
